# A Review of Economic Models Submitted to NICE’s Technology Appraisal Programme, for Treatments of T1DM & T2DM

**DOI:** 10.3389/fphar.2022.887298

**Published:** 2022-05-11

**Authors:** Marie-Josée Daly, Jamie Elvidge, Tracey Chantler, Dalia Dawoud

**Affiliations:** ^1^ Division of Anesthesiology, Department of Anesthesiology, Pharmacology, Intensive Care and Emergency Medicine, Geneva University Hospitals, Geneva, Switzerland; ^2^ National Institute for Health and Care Excellence (NICE), London, United Kingdom; ^3^ London School of Hygiene and Tropical Medicine, London, United Kingdom; ^4^ Faculty of Pharmacy, Cairo University, Giza, Egypt

**Keywords:** national institute for health and care excellence, type 2 diabetes mellitus, type 1 diabetes mellitus, health technology assessment, artificial intelligence

## Abstract

**Background:** In the UK, 4.7 million people are currently living with diabetes. This is projected to increase to 5 million by 2025. The direct and indirect costs of T1DM and T2DM are rising, and direct costs already account for approximately 10% of the National Health Service (NHS) budget.

**Objective:** The aim of this review is to assess the economic models used in the context of NICE’s Technology Appraisals (TA) Programme of T1DM and T2DM treatments, as well as to examine their compliance with the American Diabetes Association’s (ADA) guidelines on computer modelling.

**Methods:** A review of the economic models used in NICE’s TA programme of T1DM and T2DM treatments was undertaken. Relevant TAs were identified through searching the NICE website for published appraisals completed up to April 2021. The review also examined the associated Evidence Review Group (ERG) reports and Final Appraisal Documents (FAD), which are publicly accessible. ERG reports were scrutinised to identify major issues pertaining to the economic modelling. The FAD documents were then examined to assess how these issues reflected on NICE recommendations.

**Results:** Overall, 10 TAs pertaining to treatments of T1DM and T2DM were identified. Two TAs were excluded as they did not use economic models. Seven of the 8 included TAs related to a novel class of oral antidiabetic drugs (OADs), gliflozins, and one to continuous subcutaneous insulin infusion (CSII) devices. There is a lack of recent, robust data informing risk equations to enable the derivation of transition probabilities. Despite uncertainty surrounding its clinical relevance, bodyweight/BMI is a key driver in many T2DM-models. HbA1c’s reliability as a predictor of hard outcomes is uncertain, chiefly for macrovascular complications. The external validity of T1DM is even less clear. There is an inevitable trade-off between the sophistication of models’ design, their transparency and practicality.

**Conclusion:** Economic models are essential tools to support decision-making in relation to market access and ascertain diabetes technologies’ cost effectiveness. However, key structural and methodological issues exist. Models’ shortcomings should be acknowledged and contextualised within the framework of technology appraisals. Diabetes medications and other technologies should also be subject to regular and consistent re-appraisal to inform disinvestment decisions. Artificial intelligence could potentially enhance models’ transparency and practicality.

## Introduction

Diabetes mellitus (DM) is a chronic disease whose global prevalence has steeply increased over the past 4 decades, rising from 108 million in 1980 to 422 million in 2014, according to the World Health Organisation (WHO) (World Health Organisation, 2021). The disease’s global financial burden is substantial, accounting for 1.8% of the world’s Gross Domestic Product (GDP) in 2015, and likely to increase to 2.2% by 2030 (Bommer et al., 2018). DM also significantly contributes to the increase in healthcare spending in the United Kingdom (UK). In addition to DM’s rising prevalence, increased healthcare spending stem from recent and ongoing major technological innovations, developed either to treat or assist patients in the condition’s management.

In the UK, health technology manufacturers and pharmaceutical companies seeking reimbursement by the National Health Service (NHS) for their products must systematically undergo a health technology assessment (HTA). The National Institute for Health and Care Excellence (NICE) is tasked with providing coverage guidance to the NHS in England and Wales (Pearson and Rawlins, 2005). Applying companies must provide evidence of their products clinical and cost effectiveness, commonly including a cost-utility analysis (CUA), in accordance with NICE’s standards. Submissions are then thoroughly reviewed by a designated technology appraisal committee through the Technology Appraisals (TA) Programme before a final appraisal document (FAD) is published (National Institute for Health and Care Excellence (NICE), 2018).

Economic models play a critical role in the appraisal process of diabetes-specific technologies. These models are specifically designed to enable the estimation of the “hard” outcomes that are most relevant for healthcare decision making and CUA, such as mortality rates. These are usually based on extrapolation from short-term intermediate outcomes or surrogate measures, such as glycated haemoglobin (HbA1c), commonly collected in randomised controlled trials (RCTs). The choice of the model used is, however, left to the submitting companies. The technology appraisal committee is assisted by independent experts to determine whether the methodology and design are appropriate (National Institute for Health and Care Excellence (NICE), 2018). These are named evidence review group (ERG) in appraisals of single technologies (STAs) or assessment groups (AGs) in appraisals of multiple technologies (MTAs).

The objective of this study was to undertake a descriptive review of the economic models employed by submitting companies and ERGs, in the process of single or multiple TAs for the treatment of T1DM and T2DM. It was undertaken to inform ongoing work within the HTx project. HTx is a Horizon 2020 project supported by the European Union lasting for 5 years from January 2019. The main aim of HTx is to create a framework for the Next Generation Health Technology Assessment (HTA) to support patient-centred, societally oriented, real-time decision-making on access to and reimbursement for health technologies throughout Europe. The review compiled secondary data provided by the ERG and FAD reports, which are all publicly accessible once the TA process is completed and NICE guidance is published. The goal is to detect major issues raised in the reports regarding the process of the economic evaluation undertaken by companies, as well as those arising from the diabetes-specific models *per se*. The review also examines the various models’ compliance with the American Diabetes Association’s (ADA) guidelines for the “computer modelling of diabetes and its complications” (American Diabetes Association Consensus Panel, 2004). To the authors’ knowledge, this is the first such review of diabetes-specific models employed for NICE’s TA Programme. Its purpose is to provide an overview of diabetes-specific models’ shortcomings, how these eventually affect decision-making and NICE recommendations, and to outline the scope for further improvement.

## Materials and Methods

We undertook a descriptive review of the economic models used in TAs for T1DM and T2DM treatments, either STAs or MTAs, and submitted by companies seeking to obtain their product’s inclusion in NICE’s recommendations. Technologies treating diabetes-related complications, such as macular oedema and retinopathy, were not included in the review. Furthermore, only current updated TAs were examined, while TAs not employing economic models in their submissions were excluded (Table 1). This is not the first review examining the methods and processes of NICE’s TA programme. In 2019, Walton *et al.* undertook a review of STAs to expose critical issues within the TA process and enhance the programme’s efficiency (Walton et al., 2019). Other reviews examined methodological aspects of TAs pertaining to the consistency of the measurement of breast cancer utility values, or the modelling of cancer patients’ survival beyond the duration of clinical trials (Rose et al., 2018; Bell Gorrod et al., 2019; Gallacher et al., 2019). However, to the authors’ knowledge, no review has yet been conducted on the diabetes-specific models employed for NICE’s TA programme.

**TABLE 1:** List of the technologies submitted to NICE for technology appraisal, for the treatment of T1DM and T2DM.

TA	Indication	Date of publica-tion	DM type	Submit-ting company	ERG/AG	Type of economic evaluation	Model type
STA 622	Sotagliflozin with insulin for T1DM in adults	12.02.20	T1	Sanofi	BMJ TAG	Cost-utility	CORE
							PRIME^a^
STA 597	Dapagliflozin with insulin for T1DM in adults	28.08.19	T1	AstraZeneca	Warwick Evidence	Cost-utility	CARDIFF
		Updated 12.02.20					
STA 583	Ertugliflozin with metformin and a dipeptidyl peptidase-4 inhibitor for T2DM in adults	05.06.19	T2	MSD Merck Sharp & Dohme AG	Warwick Evidence	Cost-minimisation	N/A
FTA 572	Ertugliflozin as monotherapy or with metformin for T2DM in adults	27.03.19	T2	MSD Merck Sharp & Dohme AG	Warwick Evidence	Cost-comparison	N/A
STA 418	Dapagliflozin in triple therapy for T2DM in adults	23.11.16	T2	AstraZeneca	Warwick Evidence	Cost-utility	CARDIFF

MTA 390	Canagliflozin, dapagliflozin, empagliflozin as monotherapies for T2DM in adults	25.05.16	T2	Janssen-Cilag, AstraZeneca,	Warwick Evidence	Cost-utility	UKPDS-OM1
							CARDIFF
				Boehringer Ingelheim LillyUK			
							ECHO-T2DM
							UKPDS-OM1
STA 336	Empagliflozin in combination therapy for T2DM in adults	25.03.15	T2	Boehringer Ingelheim	Warwick Evidence	Cost-utility	ECEM^b^
							CORE^c^
STA 315	Canagliflozin in combination therapy for T2DM in adults	25.06.14	T2	Janssen-Cilag	SHTAC	Cost-utility	ECHO-T2DM
STA 288	Dapagliflozin in combination therapy for T2DM in adults	26.06.13	T2	AstraZeneca	Aberdeen HTA Group	Cost-utility	DCEM^d^
		Updated 23.11.16		& Bristol-Myers Squibb			
							CORE^a^
STA 151	CSII for T1DM in adults and children	23.07.08	T1	Cross-industry submission	Aberdeen HTA group	Cost-utility	CORE

a For comparison and validation.

b *de novo* model.

c For revised submission.

d Variant of the CARDIFF model.

As TA reports are listed on NICE’s website (https://www.nice.org.uk/) in open access, documents for the review were identified and downloaded in April 2021. The search was repeated in March 2022, but no new TAs were listed. Relevant TAs were accessed via the tab “NICE > NICE Guidance > Conditions and Diseases > Diabetes and other endocrinal, nutritional and metabolic conditions > Diabetes” and selected by applying the filters “technology appraisal guidance” and “published” status. TAs pertaining to the treatment of diabetic retinopathy and macular oedema were discarded. The exhaustivity of the TAs included with regard to the scope of the review was validated with NICE. The main sources of evidence consisted of secondary data, namely reports produced by the ERGs or AGs (in MTAs), and the TA committees’ FADs. The ERG reports and FAD documents for each TA were read by a single reviewer. ERG reports provide a thorough evaluation of companies’ submissions and their compliance with NICE’s guidelines on the conduct of CUA. ERGs comprehensively assess the appropriateness of the clinical effectiveness data provided, as well as critically appraise the economic modelisation performed by the companies. ERGs may also repeat part or all of the analyses on their own initiative if deemed necessary, or if compelled to do so by the committee, such as for MTAs. ERG reports sometimes included additional follow-up documents which were also examined, as a consequence of requests to the companies to provide further clarifications of their submissions. The FAD documents were then reviewed to assess how the issues raised in the ERG reports were addressed by the TA committees, and how they were reflected in NICE’s recommendation.

Although models were often comprehensively described in ERG reports, companies submissions were in some instances also reviewed to clarify certain elements of their design. Moreover, further clarifications were sometimes collected in documents published by the model developers in subsequent peer-reviewed journal articles. ERG reports regularly cited findings on models’ performance at the different editions of the Mount-Hood Challenge. The Mount-Hood Challenge network is a bi-annual conference dedicated to the computer modelling of diabetes, and meetings regularly focus on the topics of external validity and transparency. FAD documents, ERG and AG reports and company submissions are in the public domain once the recommendations are published. Therefore, no ethical approval was warranted to perform this review (London School of Hygiene and Tropical Medicine MSc Ethics Ref. 26092).

## Results

### Type of Treatment Appraised and Models Employed

Ten current TAs dealt with the treatment of T1DM and T2DM *per se* (Table 1). Two contained cost-comparison and cost-minimisation analyses only (TA572, 583) [National Institute for Health and Care Excellence (NICE), 2019a; National Institute for Health and Care Excellence (NICE), 2019b; National Institute for Health and Care Excellence (NICE), 2019c; National Institute for Health and Care Excellence (NICE), 2019d]. As these TAs did not employ economic models, they were excluded from the review. Of the 8 remaining TAs, 7 pertained to gliflozins, a new class of OAD effective for both T1DM and T2DM. In the case of T2DM, gliflozins were assessed each as single, double or triple therapy for adults and compared to the standard of care. Some molecules, such a dapagliflozin, were evaluated in several TAs for T1DM and T2DM indications, or as various lines of therapy (14–17). An MTA was performed in one case to jointly evaluate canagliflozin, dapagliflozin, and empagliflozin as monotherapies for T2DM(18, 19). Furthermore, dapagliflozin and sotagliflozin were assessed as a combination therapy with insulin in the context of T1DM. The only other class of technology appraised involved a cross-industry submission for devices procuring CSII to adults and children with T1DM, instead of multiple daily injections (20, 21). The main features of the most frequently employed models: CARDIFF, CORE, ECHO-T2DM, PRIME, UKPDS-OM1 and -OM2, are described in Table 2.

**TABLE 2:** Description of the six main models employed in NICE’s TAs, including their date of conception, their source of funding and accessibility, the type of DM encompassed, their design and simulation method, the type of software supporting them, the main source of data for outcomes, utilities, and costs.

Name	Funding	Accessibility	DM type	Model design	Simulation method	Description	Main data sources
CARDIFF	Astra-Zeneca	Via contacting developer	1 + 2	Stochastic discrete-time event	Patient-level	Programmed in C++	UKPDS (T2)
DCEM							DCCT/EDIC (T1)
2004							
ECHO-T2DM	Janssen Global Services LLC	Via contacting developer	2	Stochastic model	Patient-level	Programmed in R with Excel interface	UKPDS
2013							
IMS-CORE	Centre for Outcomes Research	Under license	1 + 2	Markov	Cohort- and patient-level	Programmed in C++	UKPDS (T2)
							DCCT/EDIC (T1)
2004							
PRIME	Eli Lilly and Company	Via contacting developer	1	Covariance matrices	Patient-level	Programmed in Java Standard Edition 8	DCCT/EDIC
2017							
UKPDS-OM1	University of Oxford	Under license	2	Probabilistic discrete-time event	Patient-level	No longer updated	UKPDS
2003							
UKPDS-OM2	University of Oxford	Under license	2	Probabilistic discrete-time event	Patient-level	Stata v12.0	UKPDS
2013							

### Major Issues Identified by ERGs

Major issues identified in the ERG reports consist of those within the companies’ modelling that are not easily rectifiable by the reviewers, or that significantly affect the incremental cost-effectiveness ratio (ICER). Major issues, specific to T1DM and T2DM models or both, are summarised in Table 3. One critical issue identified in several reports was a lack of model transparency. Indeed, the C++ interface used in the CARDIFF model was described in several reports (TA 288, 418, 597) as a “black box,” thereby restricting ERGs’ ability to cross-check or reproduce the results presented by the company [National Institute for Health and Care Excellence (NICE), 2019e; National Institute for Health and Care Excellence (NICE), 2013a; National Institute for Health and Care Excellence (NICE), 2016b]. A similar critique was made about the CORE and PRIME models (TA336, 622) [National Institute for Health and Care Excellence (NICE), 2019g; National Institute for Health and Care Excellence (NICE), 2014a]. Companies occasionally used original models, specifically designed for their product’s submission, e.g., DCEM a variant of the CARDIFF in TA288, or ECEM for TA336 [National Institute for Health and Care Excellence (NICE), 2013a; National Institute for Health and Care Excellence (NICE), 2014a; National Institute for Health and Care Excellence (NICE), 2013b; National Institute for Health and Care Excellence (NICE), 2015b]. In both cases, however, ERGs’ concern about the models’ novel design and the lack of external validity obliged companies, at the very least, to use alternative models for comparison. Furthermore, most companies favoured the use of their own sponsored models when available, such as AstraZeneca and the CARDIFF model (TA288, 390, 418 and 597).

**TABLE 3:** Summary of the major issues, specific to T1DM and T2DM models or both, identified in the ERG and AG reports.

	Major issues	Description
T1DM-models	Limited external validity proficiency	Most T1DM-models were designed using the same database, namely DCCT/EDIC, and have undergone fewer external validity reviews
	Uncertainty over models’ long-term predictive accuracy	T1DM-models have demonstrated limited accuracy at predicting hard outcomes beyond 10 years
	Limited availability of T1DM specific risk equations	The limited availability of robust clinical data to derive T1DM-specific risk equations compels the use of T2DM risk equations to predict certain vascular complications
	Unsuitability for modelling children	Despite T1DM occurring in childhood, none of the models have been designed/validated to include children
	Insufficient range of events	Some complications, preponderant to T1DM, such as DKA-related mortality and cognitive impairment associated with hypoglycaemia, are insufficiently captured or omitted in models. This ensues from a lack of robust clinical data to incorporate these outcomes
	Quality of life	QALYs may insufficiently capture the psychological or cognitive impact of certain adverse events, such as “fear of hypoglycaemia” or “cognitive impairment,” as the repercussions are less tangible than physical sequalae
T2DM-models	Limited external validity proficiency	T2DM-models predictive accuracy has not been robustly tested beyond 5–10 years. Their accuracy is also variable according to the type of complication
	Uncertainty over the predictive accuracy of certain key drivers	HbA1c has limited accuracy at predicting macrovascular complications
		Bodyweight/BMI
		- Insufficient clinical data to ascertain the sustainability of bodyweight losses associated with gliflozins
		- Insufficient clinical data to link bodyweight/BMI with hard outcomes
		- Bodyweight/BMI is sometimes a key driver although this is questionable from a clinical standpoint
All	Lack of transparency and reproducibility	Several Models described as “black boxes”, with limited ability to cross-check results despite using the same inputs
	Uncertainty over the predictive accuracy of risk equations	Risk equations might not accurately reflect outcomes in the general population, as patients with certain comorbidities were excluded from the RCTs which risk equations (e.g., UKPDS) are derived from
		As patients in RCTs with deteriorating glycaemic control would have undergone treatment intensification, risk equations double-count the effects of therapy escalation
	Limited scope of external validity appraisals	External validity appraisals do not ascertain their reliability at predicting overall costs or QALYs

Certain surrogate measures, such as HbA1c or bodyweight/BMI were identified as “key drivers” of cost effectiveness in models. However, there were methodological inconsistencies in how bodyweight/BMI influenced “hard” outcomes. Indeed, in some instances, weight affected the probability of heart failure and indirectly, the occurrence of stroke and myocardial infarction (TA622) [National Institute for Health and Care Excellence (NICE), 2019g]. In other models, weight changes only affected health-related quality of life (HRQoL) and costs (TA597) [National Institute for Health and Care Excellence (NICE), 2019e]. In fact, in most TAs, it was specifically the HRQoL associated with bodyweight change that was the key driver (TA288, 390, 418, 597) [National Institute for Health and Care Excellence (NICE), 2019e; National Institute for Health and Care Excellence (NICE), 2015a; National Institute for Health and Care Excellence (NICE), 2013a; National Institute for Health and Care Excellence (NICE), 2016b]. This finding applied to both T1DM- and T2DM-models. Thus, ICERs were sensitive to assumptions about the long-term evolution of bodyweight/BMI beyond RCTs. Yet, this was also an area of major uncertainty.

Although HbA1c *a priori* seems more legitimate than bodyweight as a key driver of model outcomes, reviewers nonetheless highlighted its limited reliability as a predictor of complications, such as macrovascular complications in T2DM(TA288) [National Institute for Health and Care Excellence (NICE), 2013a]. They also noted that certain models, such as CORE or CARDIFF, displayed limited accuracy in predicting complications, such as stroke or myocardial infarction, in external validation tests, explicitly referring to the fourth Mount-Hood Challenge (Computer modeling of diabetes and, 2007). Moreover, an ERG emphasised that the various Mount-Hood Challenges only evaluated models’ precision at predicting hard outcomes, but did not provide insights into the accuracy of predictions over costs and quality-adjusted life years (QALYs). In addition, as with bodyweight, assumptions over the medium- and long-term sustainability of HbA1c reductions beyond the duration of the RCTs was a critical area of uncertainty. ERGs questioned the reliability of UKPDS (United Kingdom Prospective Diabetes Study) risk equations to link surrogate measures and hard outcomes. They reminded the TA committee that patients over the age of 65 or with certain comorbidities, such as angina, were actually excluded from the trial from which risk equations were derived (TA288) [National Institute for Health and Care Excellence (NICE), 2013a]. Furthermore, UKPDS risk equations already incorporate a treatment escalation *per se*, as patients included in the trial with deteriorating parameters, such as HbA1c, would have undergone an intensification of their treatment. Thus, models based on UKPDS risk equations inherently double-count the treatment escalation effects (TA390) [National Institute for Health and Care Excellence (NICE), 2015a].

Some reports brought to light certain significant practical issues. In TA597, the ERG reflected on whether the additive or multiplicative approach was best suited to combine utility decrements when complications eventually accumulated, and emphasised its significant impact on the ICER [National Institute for Health and Care Excellence (NICE), 2019e]. Some models, such as PRIME, accommodate both options. The ERG considered that the best approach depended on the source of utility inputs, although the rationale for using either method should be clearly stated. In other instances, with the ECEM model (TA315), the ERG criticised the company’s choice to apply the lowest utility value among concurrent complications [National Institute for Health and Care Excellence (NICE), 2014b]. Yet the ECEM model was eventually rejected for other motives, and the method was not applied to the CORE model for the second submission.

Regarding T1DM, ERGs emphasised the importance of carrying out CUAs with dedicated models, which was the case in all three submissions. Nevertheless, reviewers highlighted the presence of gaps in data compelling the use of T2DM risk equations in some cases to model complications (TA151) [National Institute for Health and Care Excellence (NICE), 2007]. They also noted the limited evidence available to support these models’ external validity, contrary to the ADA guidelines, sometimes requiring the use of a second model to corroborate the results (TA622) [National Institute for Health and Care Excellence (NICE), 2019g]. Moreover, none of these models have been designed or have the suitable data to include children in their simulations. This was described as particularly problematic in TA151, as the CSII devices were also aimed at the under 12 age-group [National Institute for Health and Care Excellence (NICE), 2007]. Furthermore, ERGs outlined models’ lack of accuracy beyond 10 years in external validation exercises, with the CORE model in particular, displaying poor survival estimation at 25 years for T1DM, predicting 68.8% overall survival against 81.0% in the actual clinical study [National Institute for Health and Care Excellence (NICE), 2007; Palmer et al., 2004]. Moreover, the ERG in TA151 outlined limitations in the ability of QALYs to accurately capture the psychological or cognitive impact of certain adverse events, such as “fear of hypoglycaemia” or “cognitive impairment,” as the repercussions are less tangible than physical sequalae. Nevertheless, their impact especially on T1DM patients’ quality of life might be meaningful [National Institute for Health and Care Excellence (NICE), 2007]. Cognitive impairment associated with hypoglycaemia was also deemed particularly relevant to children. The ERGs believed that these aspects could significantly impact the ICER. Instead, models only accounted for the very transient utility decrement associated with hypoglycaemic episodes [National Institute for Health and Care Excellence (NICE), 2007]. The ERG in TA597 highlighted the gap in reliable data to inform utility decrements and mortality associated with diabetic ketoacidosis, which is more prominent for T1DM, although the outcome was acknowledged as very uncommon [National Institute for Health and Care Excellence (NICE), 2019e].

With regard to the final ICER for several of the T2DM TAs, ERGs reported that the results were very “unstable” as the difference in terms of overall QALY gains and costs compared to all the studied comparators were often minimal (TA315, 336, 418) [National Institute for Health and Care Excellence (NICE), 2016b; National Institute for Health and Care Excellence (NICE), 2014a; National Institute for Health and Care Excellence (NICE), 2014b]. This observation was not, however, specific to a certain type of model as three models were concerned: ECHO-T2DM, IMS-CORE and CARDIFF. Once again, this finding lead the ERGs to emphasise the critical importance of the assumptions arising from persistent areas of uncertainty and underlying each model.

### Impact of Model Limitations on Decision-Making

Most models used by companies were eventually accepted for decision making by the TA committees, with the noteworthy exception of ECEM (TA336), for which the ERG had highlighted numerous conceptual flaws [National Institute for Health and Care Excellence (NICE), 2014a; National Institute for Health and Care Excellence (NICE), 2015b]. Prior use in NICE appraisals seemed to play a significant part in the committees’ perception of models’ reliability, and despite ERGs sometimes stressing models’ lack of transparency, such as CORE and CARDIFF (TA288, 336, 597, 622) [National Institute for Health and Care Excellence (NICE), 2019f; National Institute for Health and Care Excellence (NICE), 2019h; National Institute for Health and Care Excellence (NICE), 2013b; National Institute for Health and Care Excellence (NICE), 2015b]. FAD documents also considered models’ proficiency in terms of external validity, explicitly referring to their performance in external validation assessment, such as the Mount-Hood Challenges. In the case of T1DM, and despite companies confirming their results with alternative models, FAD documents did acknowledge CORE, CARDIFF-T1DM and PRIME models’ lack of external validity, as these were built essentially on the same database, namely “Diabetes Control and Complications Trial” and “Epidemiology of Diabetes Interventions and Complications study” (DCCT/EDIC) (TAs151, 597, 622) [National Institute for Health and Care Excellence (NICE), 2019f; National Institute for Health and Care Excellence (NICE), 2019h; National Institute for Health and Care Excellence (NICE), 2008]. Committees emphasised their preference for extracting inputs from a congruent data source when feasible (TA597), or employing the more recent versions of models when available, such as the updated version of the CARDIFF model using UKPDS-82 risk equations and UKPDS-84 costs (TA418) [National Institute for Health and Care Excellence (NICE), 2019f; National Institute for Health and Care Excellence (NICE), 2016c].

When different scenarios with discrepant results were presented by companies or ERGs/AGs, the committee turned to clinical experts to ascertain which scenario was more likely to reflect reality. The clinical experts’ role was particularly critical in selecting the most appropriate scenario to predict the evolutions in surrogate measures, such as HbA1c (TA622) and BMI/weight (TA390), beyond the RCTs’ duration [National Institute for Health and Care Excellence (NICE), 2019h; National Institute for Health and Care Excellence (NICE), 2016a]. Indeed, these parameters were key drivers in many of T2DM-models as they had a significant impact on the ICER. FAD documents confirm that committees were made aware of the uncertainty surrounding HbA1c reliability in predicting macrovascular complications for T2DM, yet no alternatives were discussed. The use of bodyweight/BMI as a surrogate measure to propagate hard outcomes seemed to generate controversy. Although bodyweight/BMI did affect the onset of macrovascular complications in some models, TA622’s FAD report emphasised the insufficient evidence to corroborate this [National Institute for Health and Care Excellence (NICE), 2019h]. The committee did, however, validate this parameter’s impact on quality of life.

The committees also solicited experts’ advice on appraising the reliability and adequacy of the clinical effectiveness data used to populate the models or the risk equations employed. Furthermore, they assisted the committees in defining the best line of therapy sequence, when the HbA1c threshold of 7.5% set by NICE was exceeded, thereby warranting treatment escalation (TA390) [National Institute for Health and Care Excellence (NICE), 2016a]. Moreover, in the case of TA151, experts stressed the benefit provided by CSII in alleviating the specific needs of children under the age of 12 in terms of practicality [National Institute for Health and Care Excellence (NICE), 2008]. This acknowledgement was made despite T1DM-models not being suited to predict outcomes in children and in spite of the lack of comprehensive clinical effectiveness data in this population. The technology was nonetheless eventually recommended in the under 12 under certain conditions. Experts’ feedback to the committee were also useful to pinpoint the clinical importance of certain adverse events, insufficiently captured in models because of their very short duration. Indeed, brief events, such as hypoglycaemia, incur a very small one-off utility decrement, with only a marginal impact on QALYs (TA151) [National Institute for Health and Care Excellence (NICE), 2008]. However, such events also have significant psychological effects, referred to as “hypoglycaemic fear,” that may place an additional burden on patients’ quality of life, in addition to discouraging their adherence to treatment. In fact, TA151’s TA committee argued that part of the improvements in HbA1c observed in clinical trials for patients receiving CSII could ensue from the fewer hypoglycaemic episodes sustained, and henceforth, their enhanced therapeutic compliance [National Institute for Health and Care Excellence (NICE), 2008].

All eight reviewed submissions eventually received a positive approval from the committees in the FAD report. However, uncertainties surrounding the CUA were mitigated with the establishment of criteria in NICE’s recommendations restricting the access to the new technologies to certain patients. Uncertainties might also have led to the conclusion of confidential pricing agreements between companies and payers, which FAD documents do not report on.

## Discussion

This review examined the economic models used in published NICE appraisals of diabetes treatments. Overall, eight appraisals were included and their associated documents examined. The findings revealed that the economic models identified have a number of limitations that have repeatedly been flagged by the ERGs and appraisal committees. The key structural and methodological issues identified include the lack of external validation of model outputs, the reliance on surrogate outcomes (HbA1c and bodyweight/BMI) as predictors of hard outcomes, such as QALYs and mortality, and their pivotal influence on the final ICER estimate, as well as the reliance on outdated studies to derive the model risk equations. The review’s critical findings, its consistency with other published evidence and the implications for decision making are discussed in the following section, in addition to areas for future research.

### Differences Between T1DM- and T2DM-Models: External Validation and Transition Probabilities, Structural Uncertainty

Although T1DM and T2DM share certain characteristics, they are distinct conditions and models should preferably be designed to specifically analyse either condition. Distinct features of T1DM include younger-aged otherwise healthy patients at the onset, while T2DM is often associated with comorbidities already present at the time of diagnosis. These aspects should be reflected in the transition probabilities. However, many T1DM-models use UKPDS risk equations for certain macrovascular complications, such as stroke or chronic heart disease, which are derived from T2DM(31). This apparently stems from gaps in reliable data to derive T1DM-specific risk equations, according to model developers. Moreover, although T1DM onset is in childhood, the review reveals that no model is suited to include children or adolescents, even though some technologies, such as CSII, are aimed specifically at that population. Furthermore, some adverse events, such as cognitive impairment, that are rather specific to the consequences of hypoglycaemia among children, are not encompassed in the models’ design. Finally, the evidence supporting T1DM-models’ external validity is rather scarce, as they have been subject to fewer reviews (Computer modeling of diabetes and, 2007; The Mount Hood Six Challenge, 2012; Palmer et al., 2013; The Mount Hood 2014 Challenge, 2014; Henriksson et al., 2016; The Mount Hood 2016 Challenge, 2016; Palmer et al., 2018; Si et al., 2020).

### Cohort vs. Individual-Level Simulation Models (Transparency and Practicality)

Most of the models employed in the TA process used individual-level simulation. Microsimulation was acknowledged in both the ERG and FAD reports as a rather reliable and accurate design in comparison with cohort-simulation. ERGs did, however, report problems with some models’ computer interface, limiting their ability to cross-check or reproduce results. This observation highlights the potential trade-off between improving models’ accuracy and sophistication while also ensuring their transparency and practicality. Microsimulation models’ theoretical superiority is also debated, as one study comparing both methods did not demonstrate any systematic difference in the cost-effectiveness estimates, yet emphasised cohort models’ ease of use even for non-experts (Willis et al., 2020). The matter of transparency is particularly relevant as many pharmaceutical companies have developed their own models and could potentially skew the design and results to favour their technology. Furthermore, the review shows that most companies preferred to use their own sponsored models when available, even adjusting the design specifically for that purpose. Yet, this was met with a certain wariness, as companies were summoned by reviewers to provide additional evidence using alternative seasoned models. This emphasises the need to pursue efforts to enhance models’ transparency standards, by implementing, for example, “Diabetes Modelling Input Checklists” as proposed by the 8th Mount-Hood Challenge (Palmer et al., 2018).

### Uncertainty Surrounding the Surrogate Measures’ Accuracy in Predicting Hard Outcomes

One crucial assumption in all diabetes-specific models is their reliance on surrogate measures, notably HbA1c, to predict hard outcomes as well as diabetes-related mortality. Indeed, even very small differences in surrogate measures seem to have significant impacts on the final ICER estimates (Asche et al., 2014). This stems from the postulate that tight glycaemic control, reflected by reduction in HbA1c, should improve patients’ overall outcomes. However, evidence is accumulating against this assertion (Bejan-Angoulvant et al., 2015). HbA1c’s accuracy as a predictor of macrovascular complications or mortality is strongly disputed. An observational study in Denmark including 494 newly diagnosed T2DM patients with a 19 years overall follow-up has, for example, demonstrated that increased HbA1c levels in the first 6 years is not a good predictor of stroke, myocardial infarction, peripheral vascular disease, diabetes-related and all-cause mortality (Rozing et al., 2019). In fact, other studies have demonstrated that, although tight glycaemic control through intensive therapy does reduce microvascular complications, it also results in increase in total cardiovascular disease-related mortality and weight gain for T2DM (Ismail- Beigi et al., 2010). HbA1c’s lack of reliability as a predictor of macrovascular complications is particularly concerning, as these constitute the major cause of morbidity and mortality for patients with T2DM (Jeffcoate, 2004). The correlation between HbA1c and microvascular complications in T2DM is also debated (Bejan-Angoulvant et al., 2015). However, it is better established for T1DM(43). Some studies suggest that the variations in HbA1c could be better predictors of microvascular complications in adolescents with T1DM (Kilpatrick et al., 2008; Virk et al., 2016). Other studies have implied that time-dependent HbA1c and exponential moving average, instead of the mean HbA1c, could be more reliable to predict both type of complications (van Wijngaarden et al., 2017). Undoubtedly, more studies are needed to appraise HbA1c’s validity as a surrogate measure for T1DM and T2DM, and a proper validation of the surrogate relationship between HbA1c and hard outcomes should be undertaken.

### Reliability of Risk Equations in Modelling Transition Probabilities

Many of the models presented extract risk equations from the same data source, namely UKPDS and DCCT, to model transition probabilities (The DCCT Research Group, 1986; Adler et al., 2000; Stratton et al., 2000). However, although both studies are regarded as landmarks, they nonetheless represent a rather dated source, having been published respectively in 2000 and 1993. Risk equations are therefore likely to overestimate the occurrence of complications and mortality, as they do not reflect recent and ongoing improvements in the treatment and management of patients with T1DM and T2DM (Asche et al., 2014). Although risk equations provided by UKPDS-68 and DCCT have been updated with the subsequent collection of data from both cohorts, namely UKPDS-82 and EDIC, and are implemented in the more recent versions of several models, e.g., UKPDS-OM2 or CARDIFF for T1DM, risk equations’ accuracy is a matter of concern (The DCCT Research Group, 1986; Epidemiology of Diabetes Interventions and, 1999; Leal et al., 2013; McEwan et al., 2016). Indeed, in the case of the UKPDS study, as with many RCTs, patients included in the trial were not necessarily representative of the population living with diabetes in the UK, as individuals over 65 and those suffering from angina were excluded (Clarke et al., 2003). Furthermore, the DCCT/EDIC study was conducted in North America (The DCCT Research Group, 1986; Epidemiology of Diabetes Interventions and, 1999). Thus, the study’s results should be generalised to the UK context with caution. Another issue pertains to their redundant use in several models. This once again reflects the limited availability of robust data to derive transition probabilities, especially for T1DM. Using alternative approaches to extrapolate transition probabilities, such as linear progression or drift coefficients, may circumvent some issues associated with using risk equations, though shortcomings of the underlying data would remain.

### Weight Change as a Key Driver

This review reveals that weight is a key driver in many of the T2DM-models submitted for TA. This is despite the parameter not systematically affecting hard outcomes in models. In fact, it is the utility decrement associated with weight gain that was decisive in several TAs. However, ERG reports and FAD documents consistently outlined the uncertainty surrounding the sustainability of weight losses observed with gliflozins intake. Clinical experts also emphasised the lack of robust data supporting a correlation between weight and macrovascular or microvascular complications. The acknowledgement of weight as a key driver in models is however consistent with reviews considering other treatments and OADs, such as incretin mimetics and gliptins (DPP-4 inhibitors) (Asche et al., 2014). The review by Asche *et al.* encompassed a total of fifteen different studies with five distinct models, including CARDIFF, UKPDS-OM1&2 and CORE. Overall, eleven studies across all models identified weight as having a strong influence on the ICER (Asche et al., 2014). Although, in the case of incretin, this observation could potentially be linked to the drug’s weight reducing properties, this explanation is not plausible for gliptins as the drug has no impact on weight. However, unlike the models employed in TAs, all fifteen studies considered weight as a parameter affecting hard outcomes, with a particular emphasis on macrovascular complications, specifically congestive heart failure (Asche et al., 2014). Weight’s preponderance in models highlights the need for further research to establish whether this parameter actually affects hard outcomes, and, if so, better quantify the impact. Moreover, in the case of gliflozins, follow-up studies are required to determine whether weight losses are sustained or transient, confirming or disproving the different TA committees’ preferred assumptions.

### Lifetime Horizon

Another area of controversy is the use of long time-horizons, often lifetime, in models. This criterion is included in NICE’s reference case on the rationale that the time-horizon should suffice to capture the entirety of costs and benefits generated by the intervention [National Institute for Health and Care Excellence (NICE), 2012]. In the case of DM, this practice is justified by the significant time-lag separating the onset of complications from the initial diagnosis. Critics, however, argue that projecting surrogate measures collected in RCTs over short timeframes, into hard outcomes over decades could leave one exposed to an unreasonable level of uncertainty (Asche et al., 2014). This uncertainty is likely to persist despite varying time-horizon in a one-way deterministic sensitivity analysis. Furthermore, the external validation evaluation, such as those conducted in the different Mount-Hood Challenges rarely exceeded 5 years (Computer modeling of diabetes and, 2007; The Mount Hood Six Challenge, 2012; Palmer et al., 2013; The Mount Hood 2014 Challenge, 2014; Palmer et al., 2018; Si et al., 2020). Other external validation exercises assessing survival predictions with the CORE model in T1DM have shown a dramatic drop in accuracy beyond 10 years (Palmer et al., 2004). Divergence in predictions over time may stem from unobservable parameters, not adequately captured in the models. Although the long time-horizon is a rather intractable requirement for modelling diabetes’ long-term complications, predictions’ accuracy could nonetheless be improved with model calibration. Indeed, calibration modifies the output to adequately replicate the actual observed data used either for the internal or external validation of the model (Gray et al., 2011). Yet, experts disagree on whether calibration should be performed with external or internal data. Providing reliable calibration methods to enhance models’ long-term predictions is nevertheless an area in need of further research.

### Artificial Intelligence

The shortcomings described above highlight a need for further research and better data collection to improve the accuracy and validity of diabetes-specific models. Yet, like all models, these will nonetheless remain simplifications of reality. As outlined previously, there is a trade-off between adding to models’ complexity to improve their accuracy, while also maintaining their transparency. The abundance of data needed to feed into models could eventually overwhelm users. Applying AI to diabetes-specific models could, however, reconcile both objectives and is a promising field of research. Cognitive AI systems, in particular, could constitute a technological breakthrough for the economic modelling of diabetes and improve the predictive analytics (Hoyt et al., 2016). AI systems are likely to become capable of processing and interpreting even unstructured high volume data at speed, and can be aligned with a user-friendly collaborative interface, for example, using “R Shiny,” an open source package of the free software R, that requires minimal programming experience to use. Nonetheless, it seems that little consideration has been brought, so far, to the practical application of AI in cost-effectiveness models for diabetes. The 8th Mount-Hood Challenge has, however, purported conducting an experiment to appraise whether computer simulation models displayed elements of an intelligent learning ability, in line with Alan Turing’s proposed Turing Test (Palmer et al., 2018; Pinar Saygin et al., 2000). Yet, neither the report nor the meeting’s published proceedings reported on their findings. An ongoing case study within the HTx project is focused on developing an AI-based prediction model that can predict diabetes treatment effectiveness. The use of such prediction model can have clinical as well as methodological applications in informing future developments in diabetes economic modelling.

### Limitations of the Review

This article does not purport to be an exhaustive review of all existing diabetes-specific economic models, as its scope is limited to those used to inform NICE technology appraisals, and exclusively for interventions directly treating diabetes. Thus, economic models used in other processes, such as NICE clinical guidelines, or to treat diabetes-related complications, such as macular oedema, are not included in the review. However, the models reviewed are widely considered as the key models used in diabetes. Overall, the reviewed TAs considered only two types of technology: CSII devices, and several “me-too” drugs of a single class of OAD, i.e., gliflozins. Hence, the results of the models’ review might not be generalisable to other technologies. Moreover, this is a descriptive, not a systematic review. Therefore, the methodology is vulnerable to the risk of bias. However, we are confident that the validation of search outputs by authors from NICE improves its validity. In addition, as mentioned in the methods, although ERG reports and FAD documents are made publicly available, some specific inputs, such as costs, were sometimes not disclosed. Nonetheless, the limited access to the absolute value of certain inputs should, in practice, only marginally affect the comprehensiveness of the review as the FAD documents and ERG reports are still rather exhaustive and thorough.

Although this work does not provide a comprehensive review of all T1DM- and T2DM-models, it nevertheless provides a detailed description and analysis of some of the most commonly employed diabetes-specific economic models. The MTA of three different types of gliflozins enables one to compare and contrast the structure, assumptions and practicalities of three different models used by four different stakeholders, i.e., the three different applying pharmaceutical companies and the designated AG. The FAD document also provides detailed insights into how areas of modelling uncertainty, identified by the ERGs, subsequently influence decision-making and NICE recommendations. Moreover, as diabetes is a global concern and given that NICE is a pioneer and prominent HTA agency whose processes and methods often serve as inspirations or templates for other national agencies, this review’s results and conclusions might prove to be of value beyond the intended scope and setting.

The shortcomings of diabetes-specific economic models highlighted in this review emphasise the need to conduct further research. Specific areas for future research are summarised in Table 4.

**TABLE 4:** Areas for future research for T1DM and T2DM economic models.

Areas for future research	Description
Clinical effectiveness data	To ascertain whether weight losses or HbA1c decreases, observed with gliflozins in RCTs, are transient or sustained
	To enhance clinical effectiveness data collection among children
Bodyweight/BMI as a predictor of hard outcomes	To establish whether bodyweight/BMI changes should affect hard outcomes for T1DM, T2DM or both, and to what extent
HbA1c as a predictor of hard outcomes	To ascertain whether HbA1c is a reliable predictor of hard outcomes for T1DM and T2DM, or whether other measures, such as time-dependent HbA1c or the exponential moving average, are more accurate
Transition probabilities	To offer alternatives and updates to the UKPDS risk equations
	To develop risk equations specific to T1DM for all hard outcomes
	To robustly test the method of linear progression
Utilities and costs	To better incorporate utility decrements and costs associated with the less tangible aspects of DM, such as “fear of hypoglycaemia” or “cognitive impairment”
	To include models’ predictions of QALYs and costs in the external validity exercises
Models’ external validity	To ascertain models’ external validity beyond 5–10 years, up to a lifetime horizon
Models’ calibration	To develop reliable calibration adjusters to enhance models’ long-term predictions’ accuracy when needed
Models’ use in children	To adapt models to enable the inclusion of children
Models’ transparency and practicality	To appraise whether cognitive AI systems can be effectively incorporated into models to improve accuracy, and whether they deliver on the promise of enhancing transparency and practicality

## Conclusion

This review acknowledges that economic models help to quantify and characterise the uncertainties surrounding new technologies’ clinical and cost effectiveness, thus providing valuable support to informed decision-making. Nonetheless, designing and building models is an iterative and complex process. Hence, the review exposes a certain number of major issues that potentially undermine models’ accuracy and reliability. It also outlines which aspects of computer modelling are in critical need of further research and improvement.

To conclude, although economic models are essential tools to support decision-making and ascertain diabetes technologies’ clinical and cost effectiveness, key structural and methodological issues nevertheless remain. Thus, models’ shortcomings should be taken into consideration as well as contextualised within the framework of TAs. This also reinforces the need for TA agencies to proceed with regular and consistent technology re-appraisals, with the aim of exploiting potential updates in clinical effectiveness data, or substantial improvements in diabetes-specific economic models’ structure and design.

## Glossary

ADA: American diabetes association
AG: assessment group
AI: artificial intelligence
BMI: body mass index (kg/m2)
CUA: cost-utility analysis
DCCT: diabetes control and complications trial
DCEM: dapagliflozin cost-effectiveness model
DM: diabetes mellitus
ECEM: empagliflozin cost-effectiveness model
ECHO-T2DM: the economic and health outcomes model of type 2 diabetes mellitus
EDIC: epidemiology of diabetes interventions and complications study
ERG: evidence review group
FAD: final appraisal determination
GDP: gross domestic product
HbA1c: glycated haemoglobin blood test
HRQoL: health-related quality of life
HTA: health technology assessment
ICER: incremental cost-effectiveness ratio
MTA: multiple technology assessment
N/A: not applicable
NHS: national health service
NICE: national institute for health and care excellence
OAD: oral antidiabetic drug
QALY: quality-adjusted life-years
RCT: randomised control trial
STA: single technology assessment
T1DM: type 1 diabetes mellitus
T2DM: type 2 diabetes mellitus
TA: technology appraisal
UK: United Kingdom
UKPDS: United Kingdom prospective diabetes study
WHO: world health organisation
